# University of Global Health Equity’s Contribution to the Reduction of Education and Health Services Rationing

**DOI:** 10.15171/ijhpm.2017.56

**Published:** 2017-05-29

**Authors:** Agnes Binagwaho

**Affiliations:** ^1^Harvard Medical School, Boston, MA, USA.; ^2^Geisel School of Medicine, Dartmouth University, Hanover, NH, USA.; ^3^University of Global Health Equity, Kigali, Rwanda.

**Keywords:** Universal Health Coverage, Human Right to Health, Health Education, Health Services, Equity, Demand-Side, Supply-Side, Rural-poor

## Abstract

The inadequate supply of health workers and demand-side barriers due to clinical practice that heeds too little attention to cultural context are serious obstacles to achieving universal health coverage and the fulfillment of the human rights to health, especially for the poor and vulnerable living in remote rural areas. A number of strategies have been deployed to increase both the supply of healthcare workers and the demand for healthcare services. However, more can be done to improve service delivery as well as mitigate the geographic inequalities that exist in this field.

To contribute to overcoming these barriers and increasing access to health services, especially for the most vulnerable, Partners In Health (PIH), a US non-governmental organization specializing in equitable health service delivery, has created the University of Global Health Equity (UGHE) in a remote rural district of Rwanda. The act of building this university in such a rural setting signals a commitment to create opportunities where there have traditionally been few. Furthermore, through its state-of-the-art educational approach in a rural setting and its focus on cultural competency, UGHE is contributing to progress in the quest for equitable access to quality health services.

## Introduction

The inadequate supply of human resources in healthcare and their quality are often overlooked when discussing universal healthcare (UHC); however, this deficiency remains a significant barrier to the fulfillment of the universal right to health.^1,2^ Without a serious commitment to improving the quantity and quality of health service providers, UHC will remain an unattainable goal due to the rationing of health services.^3^ In many low-income countries, where the majority of the population lives with limited resources, access to health services is a chronic challenge for financial reasons. When the capacity to deliver needed services is lacking, the poor and the vulnerable are often the most affected.^4-6^ This paper seeks to discuss why Partners in Health (PIH) – a US based non-governmental organization – has decided to create an institution of higher education in rural Rwanda, known as the University of Global Health Equity (UGHE). By changing both *where* and *how* health education is provided, UGHE will contribute to reducing the health workforce gaps that disproportionately and detrimentally affect the poor. The approach of this new health sciences university, if replicated will also help to improve the distribution of health service providers, allowing for better access to health services for the population as a whole, especially the poor and vulnerable.

## Supply of Services

All countries, rich and poor, are concerned with staffing their health systems, and have gaps in the quantity, quality, and distribution of their health workforce.^1^ These gaps are most acute in developing countries, and further exacerbated by the within-country inequities that persistently exclude the poorest segments of the population.^4^ Efforts to address these inequities have been made by some countries, but the poor remain under-served. Even with initiating “pro-poor” health system reforms, improving the distribution of the health workforce, increasing investments in the health sector, as well as designing of strong legal framework for UHC, the problem is far from being resolved.^4^

Many strategies are being deployed to reduce barriers to this supply issue, with some of the most important involving the education of non-physician clinicians and task-shifting.^7,8^ New initiatives have been developed to increase the number of healthcare providers. The Human Resource for Health (HRH) program led by the Ministry of Health of Rwanda, in partnership with 23 American institutions of higher education is a successful, innovative example of how to address these healthcare workforce challenges.^9^ The HRH project aims to support the University of Rwanda and Rwandan teaching hospitals by increasing their teaching capacity in medicine, nursing and dentistry.^9^ This has benefited the health sector in multiple ways, including direct improvements to the quality of services provided in Rwandan hospitals and an increase in the teaching capacity of the University - which tripled the number of medical students, as well as starting a new school of dentistry and several residency programs.^9^

Despite the success of initiatives such as the HRH program in Rwanda, solving the supply problem worldwide requires more programs that will contribute to educating health professionals. To contribute to solving this problem, UGHE was created as an institution of higher learning, accredited in Rwanda, and adopting East African and American standards to pursue international accreditation. As a health sciences university, UGHE has a clear equity agenda based on community healthcare delivery and innovations in implementation sciences. UGHE was created with the long-term vision of expanding across four continents, including the 10 countries where PIH currently operates. Rwanda is the first site of several future interconnected higher-level health education campuses to be built alongside PIH clinical service delivery sites.^10^

## Demand of Services

Demand-side barriers are equally important obstacles for people to access health services and exercise their right to health. In some developing countries, the poor and vulnerable are not only under-served because of the supply factors outlined above, but also because of their reluctance or inability to seek services.^11^

Complex, multifactorial social determinants of health constitute many of the barriers that prevent vulnerable people from accessing quality health services. These determinants vary widely by context and encompass aspects of the judicial system, race, place of birth and living, environmental conditions, and more.^12^ Given the importance of interrogating these barriers, UGHE has embraced a curriculum that integrates them into each course. These determinants are related to “supply-side” factors as noted above, including the inadequate geographic distribution of health professionals. While the majority of the poor live in rural areas, they are largely underserved by trained health professionals whose preference is for working in urban areas.^11^ In remote areas, there also exists a lower demand for services among the rural poor because of the relative scarcity of financial resources and means of transportation to seek services from health professionals where they are located and working.^11^ The strategic deployment of the health workforce is key to ensuring adequate geographic coverage of health services. Creating a more equitable distribution that best meets the needs of the population, regardless of where they live, is critical. Without this, the existing geographic exclusion and rationing of access to health services will persist.^11^

There is evidence showing that educating health workers in rural areas may motivate them to remain and work there.^13,14^ This explains why PIH decided to build the main campus of UGHE in Burera District, a remote area in the Northern province of Rwanda. By receiving the majority of their training and mentorship from professionals and educators who serve Rwandans in a rural settings, a new generation of health services providers will be purposely and strongly sensitized to serve rural populations in the future.^13,15^ In doing this, UGHE applies a “pro-poor” strategy that facilitates the retention of qualified health professionals in remote rural areas. If the example of PIH is emulated and spread elsewhere, it has the potential to increase access to health services for the vulnerable and geographically isolated.

Another demand-side barrier to care-seeking involves the lack of consistent, patient-centered attitudes of some healthcare providers.^11^ While many clinicians work with a professional, patient-centered dedication, there are too many that fail to listen as attentively or respectfully as their patients deserve, and sometimes fail to analyze their situation in a humane and comprehensive manner.^11^ In clinical practice, cultural and contextual dimensions are often not given the importance they deserve during interactions between poor patients and healthcare providers.^11^ Further, an imbalanced power dynamic that can be exacerbated by socio-economic differences between patient and provider can contribute to patients’ unease and the feeling that they are not truly being listened to within the clinical setting.^4^ This creates conditions that lead some patients to avoid seeking healthcare services, even when they are available.^11^ The lack of cultural sensitivity and compassion among health workers is a systemic global problem.

Unfortunately, too few institutions of higher education train health professionals to provide quality, comprehensive services with an attention to the cultural context, compassionate listening and empowering communication at all points of the continuum of care.^4^ The way curricula are conceived and taught is at the core of this problem,^16^ which leads to health professionals lacking these vital care-giving skills. The importance of medical education in shaping the mindset of its trainees cannot be overstated.^17^ As such, it is necessary to rethink how healthcare provider education is designed to address these gaps and produce needed transformation in this domain. If these important changes are not made, we are accepting a “rationing of health sciences education” – a parallel to “health rationing” – which produces health professionals ill-equipped to serve the needs of their patients.^17^ Arguably, health science education rationing can be translated into healthcare rationing and serve as a barrier to patients fully experiencing their right to health.

As such, it is necessary to develop a new type of educational system in healthcare, which deliberately and actively focuses on delivering equitable health services with cultural competency to all including people from rural areas and poor settings. This approach to education is similar to the battle for social justice that took place nearly two decades ago with the rationing of HIV services. That battle facilitated task-shifting efforts to ensure better access to HIV clinical services for the poor in low-income countries.^18^

As part of the solution to this challenge, UGHE proposes a different approach to education that focuses on increasing the demand for services. Clinicians will be educated in comprehensive care, which considers all the dimensions of sickness, illness and health. Further, these students will be trained and equipped with biosocial analysis tools to provide quality services for all. The UGHE curriculum will be focused on ensuring patients are approached not as a sick organ or disease but rather as human beings, within a complex environment. UGHE has already introduced significant educational reforms to achieve these goals. For example, all graduates will obtain dual degrees – a Master in the Science of Health Services Delivery will be obtained along with a clinical degree. Upon admission, doctors, nurses and all other clinicians at the university, will be engaged in a multidisciplinary learning environment inside and outside the classroom, receiving the specific training for their field only when called for. Every year they will have to complete a community practicum in a local village, first experiencing work as a Community Health Worker (CHW), and will be supervised by faculty members including experienced CHWs, nurses and doctors skilled in community medicine. This is just one of many ways how UGHE is working to transform healthcare education with an equity focus.

## Conclusion

Too many institutions of higher education fail to produce health professionals that completed their program with a sense of true cultural competency and commitment to equity. This is an obstacle to the attainment of UHC and the fulfillment of the right to health for all. To promote the human right to health, all the components of UHC should be considered as they contribute implicitly and/or explicitly to rationing access to clinical services. To reduce the supply and demand barriers to health services, there needs to be a renewed focus on better geographically distributed trained clinicians so that the poor and vulnerable are not left behind. More institutions need to revisit their mission and critically evaluate how they can better equip their future healthcare professionals with the cultural competencies needed to provide quality care. It is the hope that PIH’s example in creating UGHE can serve as a catalyst for this reflection as we aim to further advance this agenda for equity.

## Ethical issues

Not applicable.

## Competing interests

Author declares that she has no competing interests.

## Author’s contribution

AB is the single author of the paper.

## References

[1] World Health Organization (WHO). Working Together for Health - The World Health Report 2006. Vol 19. Geneva: WHO; 2006.

[2] World Health Organization (WHO). Transforming and Scaling up Health Professionals’ Education and Training: WHO Education Guidelines 2013. Geneva: WHO; 2013. 26042324

[3] World Health Organization. Global Strategy on Human Resources for Health: Workforce 2030. http://www.who.int/hrh/resources/pub_globstrathrh-2030/en/. Published 2016.

[4] Kim JY, Farmer P, Porter ME (2013). Redefining global health-care delivery. Lancet.

[5] The World Bank. Poverty and Health. World Bank Briefs. http://www.worldbank.org/en/topic/health/brief/poverty-health. Accessed March 25, 2017. Published 2014.

[6] Braveman P, Gruskin S (2003). Poverty, equity, human rights and health. Bull World Health Organ.

[7] Binagwaho A, Sarriera G, Eagan A (2017). The evolution of the physician role in the setting of increased non-physician clinicians in sub-Saharan Africa: an insistence on timing and culturally-sensitive, purposefully selected skill development: Comment on “Non-physician clinicians in sub-Saharan. Int J Health Policy Manag.

[8] Shumbusho F, Van Griensven J, Lowrance D (2009). Task shifting for scale-up of HIV care: Evaluation of nurse-centered antiretroviral treatment at Rural Health Centers in Rwanda. PLoS Med.

[9] Cancedda C, Riviello R, Wilson K, et al. Building workforce capacity abroad while strengthening global health programs at home: participation of seven Harvard-affiliated institutions in a Health Professional Training Initiative in Rwanda. Acad Med 2017; Forthcoming. 10.1097/ACM.0000000000001638 28328735

[10] PIH Countries. Partners In Health website http://www.pih.org/countries. Accessed May 1, 2017. Published 2017.

[11] Abiiro GA, Mbera GB, De Allegri M (2014). Gaps in universal health coverage in Malawi: a qualitative study in rural communities. BMC Health Serv Res.

[12] WHO. Social determinants of health. http://www.who.int/social_determinants/en/. Accessed May 1, 2017. Published 2017.

[13] Woloschuk W, Tarrant M (2002). Does a rural educational experience influence students’ likelihood of rural practice? Impact of student background and gender. Med Educ.

[14] Dossajee H, Obonyo N, Ahmed SM (2016). Career preferences of final year medical students at a medical school in Kenya--A cross sectional study. BMC Med Educ.

[15] Hurst SA (2014). Eroding students’ rural motivation: First do no harm?. Swiss Med Wkly.

[16] Teichholtz S, Kreniske JS, Morrison Z, Shack AR, Dwolatzky T (2015). Teaching corner: an undergraduate medical education program comprehensively integrating global health and global health ethics as core curricula: student experiences of the medical school for international health in Israel. J Bioeth Inq.

[17] Schmidt VH (2004). Models of Health Care Rationing. Curr Sociol.

[18] Price J, Binagwaho A (2010). From medical rationing to the use of human resources for AIDS care and treatment in Africa: a case for task shifting. Dev World Bioeth.

